# Urokinase-type plasminogen activator deficiency enhances CD8^+^ T cell infiltration and anti-PD-1 therapy efficacy in prostate cancer

**DOI:** 10.3389/fimmu.2025.1625226

**Published:** 2025-09-01

**Authors:** Xiaoyi Li, Xiao Zhang, Xing Fu, Hong Wu, Xinyu Ye, Xin Huang, Yuhao Cui, Chao-Nan Qian, Yi Lu, Jian Zhang

**Affiliations:** ^1^ Department of Human Cell Biology and Genetics, School of Medicine, Southern University of Science and Technology, Shenzhen, Guangdong, China; ^2^ Infectious Disease Center, Guangzhou Eighth People's Hospital, Guangzhou Medical University, Guangzhou, Guangdong, China; ^3^ Department of Pharmacology, Molecular Cancer Research Center, School of Medicine, Sun Yat-sen University, Shenzhen, Guangdong, China; ^4^ Department of Radiation Oncology, Guangzhou Concord Cancer Center, Guangzhou, Guangdong, China; ^5^ Joint Laboratory of Guangdong-Hong Kong Universities for Vascular Homeostasis and Diseases, SUSTech Homeostatic Medicine Institute, School of Medicine, Southern University of Science and Technology, Shenzhen, Guangdong, China; ^6^ Clinical Research Center, The First People's Hospital of Foshan (Affiliated Foshan Hospital of Southern University of Science and Technology), School of Medicine, Southern University of Science and Technology, Foshan, Guangdong, China

**Keywords:** urokinase-type plasminogen activator, prostate cancer, CD8^+^ T cells, tumor immune microenvironment, combination therapy

## Abstract

**Introduction:**

Urokinase-type plasminogen activator (uPA) is upregulated in prostate cancer, but its comprehensive impact on the immune microenvironment and the underlying mechanisms remains to be fully elucidated.

**Methods:**

uPA expression was analyzed in clinical prostate cancer specimens and correlated with CD8⁺ T cell infiltration. Tumor growth was assessed in the uPA-deficient (uPA^–/–^)and the uPA inhibitor UK122-treated mouse model. Immune infiltration was evaluated by CyTOF and flow cytometry. Anti-CD19 chimeric antigen receptor (CAR)-engineered WT or uPA^–/–^ CD8⁺ T cells were tested for cytotoxicity against RM1-CD19 cells. The combination of UK122 and anti-PD-1 therapy was assessed.

**Results:**

Elevated uPA in prostate cancer specimens inversely correlated with CD8⁺ T cell infiltration. Both genetic uPA ablation and UK122 significantly attenuated tumor growth by enhancing antitumor immunity. uPA deficiency markedly increased CD8⁺ T cell infiltration. uPA^–/–^ CD8⁺ T cells exhibited enhanced cytotoxicity compared to WT CD8⁺ T cells. Tumor-infiltrating uPA^–/–^ CD8⁺ T cells showed higher PD-1 expression. UK122 synergized with anti-PD-1 therapy to promote tumor regression.

**Discussion:**

uPA is a significant immunosuppressive regulator in prostate cancer. Its inhibition enhances CD8⁺ T cell function and synergizes with immune checkpoint blockade, supporting uPA targeting as a novel strategy to improve prostate cancer immunotherapy efficacy.

## 1 Introduction

Prostate cancer is the most diagnosed cancer in men globally, with approximately 1.4 million new cases in 2020 (1–3). Projections indicate approximately 1,053,250 new cancer cases in 2025, including 313,780 incident cases of prostate cancer (4). While localized disease is managed effectively through radical prostatectomy and androgen deprivation therapy, approximately 10-20% of patients eventually develop castration-resistant prostate cancer (CRPC) (5, 6), underscoring the urgent need for novel therapeutic strategies.

Immunotherapy has emerged as a transformative approach in oncology, with immune checkpoint inhibitors (ICIs) showing promise (5–7). However, prostate cancer exhibits remarkable resistance to ICI therapy, with objective response rates below 20% in unselected populations (8). This resistance derives from multiple factors, including low tumor mutational burden, elevated immune checkpoint expression, T cells exhaustion, and regulatory T cells (Tregs) (9, 10). These barriers are further compounded by the recruitment of M2 macrophages, which create an immunosuppressive microenvironment that functionally restricts CD8^+^ T cells access to tumor cells (11, 12).

CD8^+^ T cells play an essential role in the antitumor immunity by directly killing tumor cells. Higher intratumoral CD8^+^ T cells infiltration correlated with improved survival in patients after radical prostatectomy, suggesting that enhancing CD8^+^ T cells enrichment could be a potential therapeutic strategy (13). Moreover, the proximity of CD8^+^ T cells to tumor cells does not guarantee a strong immune response, as they overexpress immune checkpoint genes (ICGs) such as programmed cell death protein 1 (PD-1; encoded by the Pdcd1 gene), lymphocyte activation gene 3 protein (LAG3), and cytotoxic T-lymphocyte-associated protein 4 (CTLA4). Critically, upon binding to their respective ligands (PD-L1/PD-L2, MHC class II, and CD80/CD86) expressed on tumor or antigen-presenting cells, these receptors initiate potent immunosuppressive signaling cascades (14–16). This situation reduces the secretion of cytotoxic effector cytokines, such as Granzyme B (GzmB), interferon-gamma (IFN-γ), and tumor necrosis factor-alpha (TNF-α), which are essential for orchestrating immune responses (17, 18). This limitation is evident in clinical trials, in which ICIs have shown limited effectiveness in unselected advanced prostate cancer populations (19). Therefore, identifying new therapeutic targets is essential for improving early detection, diagnosis, and prostate cancer immunotherapy.

The urokinase-type plasminogen activator (uPA) is a serine protease that converts plasminogen to plasmin, essential for remodeling the extracellular matrix (ECM) (20, 21). The uPA–uPA receptor (uPAR) interaction activates plasminogen, enhancing plasmin production and promoting cancer cell survival, proliferation, and migration (22). This has led to the development of uPA inhibitors, such as Aprotinin, Amiloride, and UK122, which have shown effectiveness in cancer treatment by reducing plasmin production and ECM degradation, limiting tumor invasion (23–25).

In this study, our findings demonstrate increased uPA expression in prostate cancer tissues and its negative association with CD8^+^ T cells infiltration. Additionally, we further investigate the role of uPA in the tumor immune microenvironment and the combination therapy of uPA inhibitors and PD-1 blockade in prostate cancer.

## 2 Materials and methods

### 2.1 Cell culture

The murine prostate cancer RM-1 cell line and 293T were obtained from the American Type Culture Collection (ATCC). RM-1 cells and 293T were cultured in RPMI 1640 (Gibco, C11875500BT) and DMEM (Gibco, 10313021), respectively, supplemented with 10% fetal bovine serum (Gibco, A5669701) and 1× penicillin-streptomycin (BBI, E607011-0100) at 37°C under 5% CO_2_ in a humidified incubator.

### 2.2 Mice

Male wildtype C57BL/6J mice (WT, Stock No. 000664) and male uPA^−/−^ mice (Stock no. T027315) were purchased from GemPharmatech Co., Ltd. (Nanjing, Jiangsu, China). All mice (age 6–8 weeks, weight within the range of 20–25 g, 5–10 mice per group) used in this study were housed under pathogen-free conditions, with five mice per cage, and kept in the Laboratory Animal Center of Southern University of Science and Technology.

The sample size for each experiment was determined using G*Power (version 3.1.9.7) based on preliminary data and expected effect sizes. We assumed an effect size of 0.8 (max effect), a statistical power of 90%, and a significance level (α) of 0.05 for all experiments. The calculated sample size was adjusted for potential dropouts (10%) to ensure robust statistical analysis.

For all subcutaneous transplantation models used in this study, RM-1 cells (5 ×  10^5^ cells/0.1 mL of PBS) were injected subcutaneously into the back of the WT or uPA^–/–^ mice. To deplete CD8^+^ T cells, WT and uPA^–/–^ mice were randomly divided into the Anti-CD8α (WT Anti-CD8α and uPA^–/–^ anti-CD8α, 6 mice per group) groups and the isotype (WT Isotype and uPA^–/–^ Isotype) groups (6 mice per group) using a computer-based random number generator (Microsoft Excel, RAND function). The anti-CD8α group underwent CD8^+^ T cells depletion via intraperitoneal injections of anti-CD8α antibody (100 μg/mice, Selleck, A2102), while the isotype (WT Isotype and uPA^–/–^ Isotype) groups received isotype antibody (100 μg/mice, Selleck, A2116) every two days for a total of three times. For combined therapy, Thirty-two WT mice were randomly divided into four groups (8 mice per group) using a computer-based random number generator: Control (100 μl of DMSO), uPA inhibitor (4 mg/kg UK122; MedChemExpress, HY-111056), anti-PD-1(100 μg/mice; STARTER, S0B0594), combined UK122 (4 mg/kg), and anti-PD-1(100 μg/mice); intraperitoneal injection was performed every day for a total of seven times.

In the experiments described above, the tumor volume (mm^3^) was measured every 2 days from Day 2 and continued for 10–12 days. The tumor volume was calculated using the formula: volume = (length × width^2^)/2. When the experimental endpoint was reached, or if the tumor volume reached 1,500 mm^3^ or body weight loss exceeded 15% during the experiment, euthanasia was performed on the mice via cervical dislocation under anesthesia (using 1.25% avertin, 0.2 mL/10 g, intraperitoneally), with death confirmed by cessation of heartbeat. Subcutaneous tumors were then collected for further analysis, such as flow cytometry or immunohistochemistry.

All animal experimental procedures were approved by the Animal Experimentation Ethics Committee of Southern University of Science and Technology (No. SUSTech-JY202408015) and adhered to ARRIVE guidelines.

### 2.3 RNA-sequencing and analysis

Total RNA was extracted from the tumor tissues of WT and uPA^–/–^ mice, and all RNA-seq samples were quality-controlled, library preparation, and analysis by Genedenovo Biotechnology Co., Ltd. (Guangzhou, China). R version 4.4.0 was used for statistical analysis. A differential gene expression (DEGs) analysis was performed using the *DESeq2* R package, with genes with a *P*-value ≤ 0.05 and a log2 fold change of +1 or −1 considered significantly upregulated and downregulated, respectively. Gene Ontology (GO) and Kyoto Encyclopedia of Genes and Genomes (KEGG) enrichment analyses of the upregulated and downregulated genes were conducted using the *ClusterProfiler* R package; pathways with an FDR ≤ 0.05 were considered significant.

### 2.4 Immunohistochemistry analysis

Immunohistochemistry (IHC) was performed using subcutaneous tumors after 12 days of inoculation. Tumor tissues were fixed in formalin and embedded in paraffin. Immunohistochemical (IHC) analyses of CD8α, GzmB, anti-TNF-α, and IFN-γ were performed on tumors from WT and uPA^−/−^ mice, and the expression of uPA and CD8α was also analyzed in a human prostate cancer tissue microarray. The IHC results were visualized using CaseViewer 2.3, to observe. Six to eight different areas were selected from each sample, and the proportion of positive cells was calculated using the ImageJ software. A multiplex immunohistochemistry (mIHC) for PD-1^+^CD8^+^ T cells in tissue sections was performed using a TSA ^®^ Plus fluorescein detection kit (Servicebio, G1256), according to the manufacturer’s instructions. The primary antibodies were listed in **Supplementary Table 1**.

### 2.5 Establishment of the RM1-hCD19 cell line

To produce lentiviruses, 293T cells were plated in a 10-cm dish. The next day, the lipofectamine 3000 (Thermo Fisher Scientific, L3000015) was mixed with OPTI-MEM (Gibco, 31985070). A plasmid mix included 10 μg of the vector with 5 μg of both psPAX2 and pMD2G, incubated for 15 min at room temperature before gently adding the mixture to the 293T dish and swirling. At 48–72 h post-transfection, the RM-1 cells were cultured with the viral-containing supernatant for 3 days, followed by selection with puromycin for 7 days. Finally, the expression efficiency of human CD19 (hCD19) in RM1-CD19 cells was verified using flow cytometry.

### 2.6 Construction of anti-CD19 CAR

The anti-CD19-specific FMC63 chimeric antigen receptor (CAR) consists of a Flag-tagged FMC63 scFv (26), CD28, and CD3ζ intracellular domain (27). The anti-CD19 CAR sequence was cloned into the MSCV retroviral vector, followed by MSCV-CD19 CAR and MSCV (empty construct without the CD19 CAR coding sequence).

### 2.7 Retrovirus production

To produce the retrovirus, 20 µL of Mirius TransIT^®^-LT1 (Mirus, MIR 2300) was mixed with 1980 µL of pure OPTI-MEM (without FBS, P/S, or Glutamax, Gibco, 31985070) for each 10-cm dish of 293T. The plasmid mix was prepared per dish, including 2.5 μ g of pCL-Eco and 7.5 µg of retroviral vector (MSCV-CD19 CAR). The liposome solution (20 µL) was mixed and added to the plasmid mix and incubated for 15 min at room temperature before gently adding the mixture to the 293T dish and swirling. At 48 and 72 h transfections, the supernatant was collected and filtered.

### 2.8 Generation of CD8^+^CAR- T cells

To obtain activated CD8^+^ T cells, the 24-well plate was coated with anti-CD3 (Thermo Fisher, 16-0032-81) and anti-CD28 (Thermo Fisher, 16-0281-81) at a concentration of 1 µg/mL in PBS overnight at 4°C or for 2–4 h at 37°C with RPMI 1640 (10% FBS, Glutamax (Gibco, 35050061), 1× penicillin-streptomycin, and 100 µM β-mercaptoethanol (Bestbio, BB-92003)). Next, splenic CD8^+^ T cells was isolated from WT and uPA^–/–^ mice, which were resuspended at 0.5 × 10^6^ cells/mL; 2 mL was added to each well at 37°C or for 48 h.

Subsequently, a 24-well plate was coated with retronectin (Takara, T100AC; 7 µL in 1 mL of PBS) and incubated overnight at 4°C. Then, the retronectin was removed, 1 mL of the virus supernatant of MSCV-CD19 CAR was added, and the mix was centrifuged in 2000 × *g* for 1.5 h. The 0.5 × 10^6^ cells/ml of activated CD8^+^ T cells were cultured at a 24-well plate binding virus and with a medium containing IL-7 (10 ng/mL; Novoprotein, CC73) and IL-15 (20 ng/mL; Novoprotein, GMP-C016) and used within 3–4 days. Finally, the expression efficiency of anti-CD19 CAR in CD8^+^ T cells was verified using flow cytometry.

### 2.9 Cell cytotoxicity assay

The specific killing activity of CD8^+^CAR T cells against the target RM1-CD19 cells was measured using the CytoTox 96^®^ Nonradioactive Cytotoxicity Assay (Promega, G1780). This assay enables the quantification of cell lysis by measuring the release of lactate dehydrogenase (LDH) from damaged cells into the supernatant. Therefore, CD8^+^ CAR-T cells were cocultured with targeT cells (RM1-CD19) at various ratios for 24 hours. Following this incubation, the supernatants were collected, and the total cells were lysed to evaluate total LDH release. Cytotoxicity was calculated according to the manufacturer’s protocol: % Cytotoxicity = 100× (Experiment–Effector Spontaneous–Target Spontaneous)/(Target Maximum–Target Spontaneous) (28–30).

### 2.10 Flow cytometry

Single-cell suspensions were isolated from the RM-1 subcutaneous tumor. The cells were washed twice with PBS and treated with LIVE/DEAD Fixable Aqua (BioLegend, 423101). After another wash with PBS, the cells were stained with Fc-blocking antibodies against CD16/32. Then the cells were stained with surface antibodies in the dark at 4°C for 30 min. For intracellular factor stimulation, cells were cultured with a cell-stimulation cocktail containing protein-transport inhibitors (eBioscience, 00-4975-03) for 4 h at 37°C. Next, the cells were harvested, fixed, and permeabilized with 100 μL of 1× fixation buffer and permeabilization buffer (eBioscience, 88-8824). Then, the cells were stained with intracellular antibodies in the dark at 4°C for 30 min. Cell suspensions were analyzed on a BD FACSAria instrument (BD, FACSAria SORP). Data were analyzed offline using FlowJo (v. 10.8.1). The antibodies used in these experiments are listed in **Supplementary Table 2**.

### 2.11 Enzyme-linked immunosorbent assays

The cytokines in the serum from WT and uPA^–/–^ tumor-bearing mice were measured by enzyme-linked immunosorbent assay (ELISA) kits of IFN-γ (Animaluni, LV30253M), GzmB (Animaluni, LV30229), and TNF-α (Animaluni, LV30536M), following the manufacturer’s protocol.

### 2.12 Cytometry by Time-Of-Flight (CyTOF) analysis

Tumor tissues were harvested from the subcutaneous RM-1 cell tumor model and then prepared into a single-cell suspension as previously described. The protocol used for CyTOF sample preparation and acquisition was based on a previous report (31). We utilized 16 channels for the analysis, specifically targeting various cell types and functions. The antibodies and reagents used in these experiments are listed in **Supplementary Tables 3**, **4**, respectively.

### 2.13 Tissue microarray

The human prostate cancer tissue microarray used in this study was obtained from Servicebio Technology Co., Ltd. (Wuhan, China), comprising 32 paired peritumor-tumor tissues and 27 tumor-only specimens. Following rigorous quality control, which excluded samples with tissue fragmentation exceeding 50% or epithelial content loss greater than 30%, a total of 35 eligible samples (19 matched pairs and 16 tumor-only specimens) were retained for immunohistochemical analysis of uPA and CD8α.

The Medical Ethics Committee of Southern University of Science and Technology formally determined that this study did not require ethical review (Approval No. 20230132), as it utilized pre-existing, fully anonymized archival specimens with no access to protected health information, in compliance with the Declaration of Helsinki.

### 2.14 Statistical analysis

The bioinformatics analysis process performed in the study was realized through R software (4.4.0). Data are presented as the mean ± SEM and were analyzed using GraphPad Prism, version 10.1.2. A two-tailed unpaired Student’s *t*-test was determined to assess statistical significance; one-way ANOVA followed by the Tukey multiple-comparison test was performed to compare two groups. A two-way ANOVA with Dunnett’s multiple-comparison test evaluated the treatment effects on tumor growth. Normality was evaluated using the Shapiro-Wilk test, and homogeneity of variances was assessed using Levene’s test. If the data did not meet the assumption of normality, non-parametric tests such as the Mann-Whitney U test or Kruskal-Wallis test were applied instead. The cumulative survival time was estimated using the Kaplan-Meier method, and the log-rank test was utilized to compare the different groups. Correlation analysis was conducted using the Pearson method. Significance was set at *P <*0.05 (*, *P* < 0.05; **, *P* < 0.01; ***, *P* < 0.001; ****, *P* < 0.0001).

## 3 Results

### 3.1 Elevated uPA expression in human prostate cancer is negatively correlated with intratumoral CD8^+^ T cells infiltration

**Figure 1:**
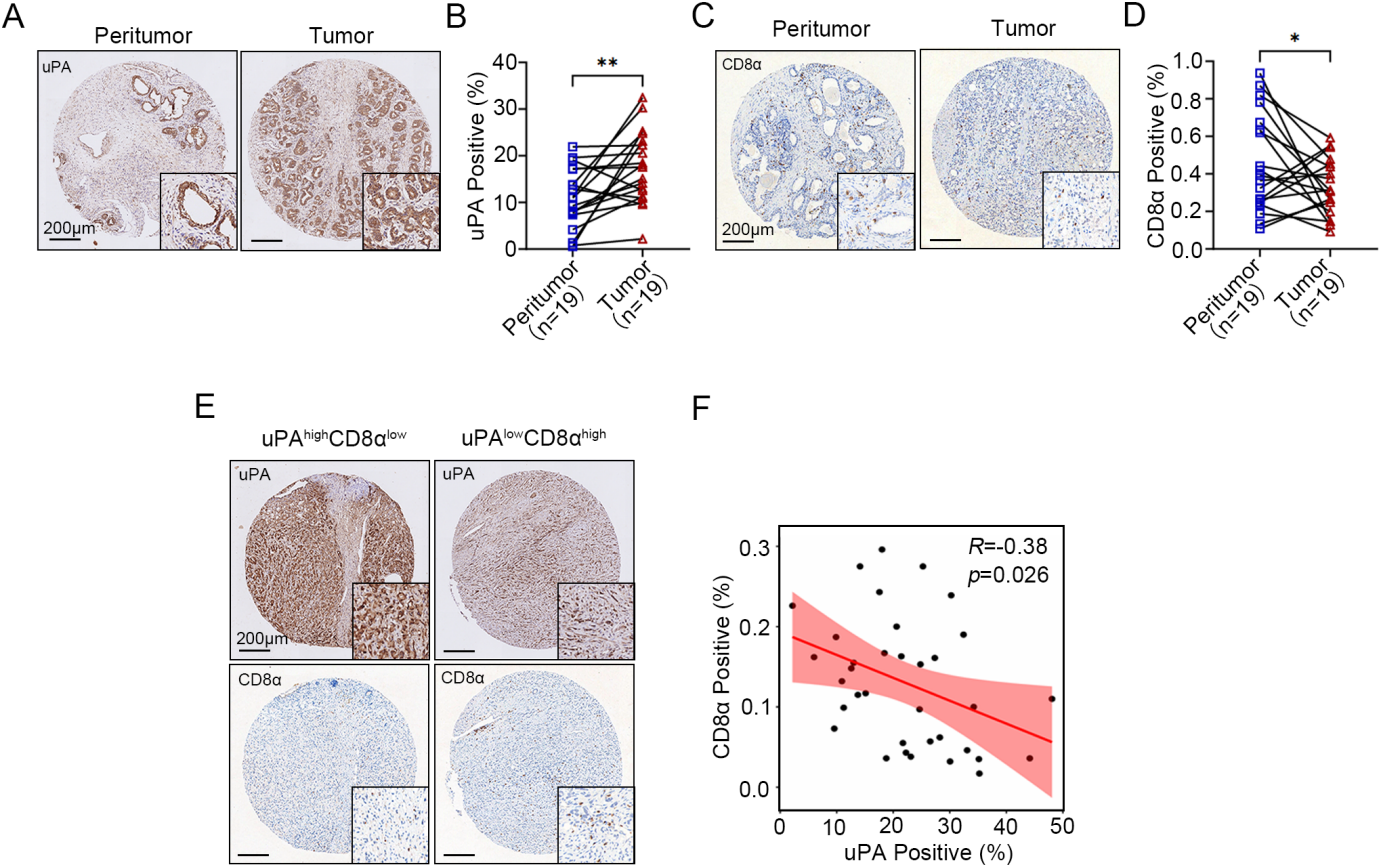
Elevated uPA expression in human prostate cancer is negatively correlated with intratumoral CD8^+^ T cell infiltration. **(A, B)** Immunostaining images and quantification of uPA expression in 19 pairs of peritumoral and tumor tissues from patients with prostate cancer (n = 19). Scale bar, 200 μm. **(C, D)** Immunostaining images and quantification of CD8^+^ T cells in 19 pairs of peritumoral and tumor tissues from patients with prostate cancer (n = 19). Scale bar, 200 μm. **(E, F)** Correlations between the expression levels of uPA and CD8α in prostate cancer tumor tissues (n = 35); scale bar, 200 μm. The results were presented as the mean ± SEM, * P < 0.05. ** P < 0.01.

To evaluate the role of uPA in human prostate cancer, we performed IHC to assess the expression of uPA in both prostate cancer and peritumoral tissues. The results showed that uPA was significantly overexpressed in prostate cancer compared to peritumoral tissues (**Figures 1A, B**, **Supplementary Figure 1A**), suggesting that uPA may be an effective therapeutic target for prostate cancer. Additionally, the infiltration of CD8^+^ T cells were decreased in prostate cancer compared to peritumoral tissues (**Figures 1C, D**, **Supplementary Figure 1B**), and the expression of uPA was negatively correlated with CD8^+^ T cells infiltration (*R* = -0.38, *P* = 0.026; **Figures 1E, F**), indicating that highly expression of uPA was associated with poorer CD8^+^ T cells infiltration in patients with prostate cancer.

### 3.2 uPA deficiency inhibits prostate cancer progression

**Figure 2:**
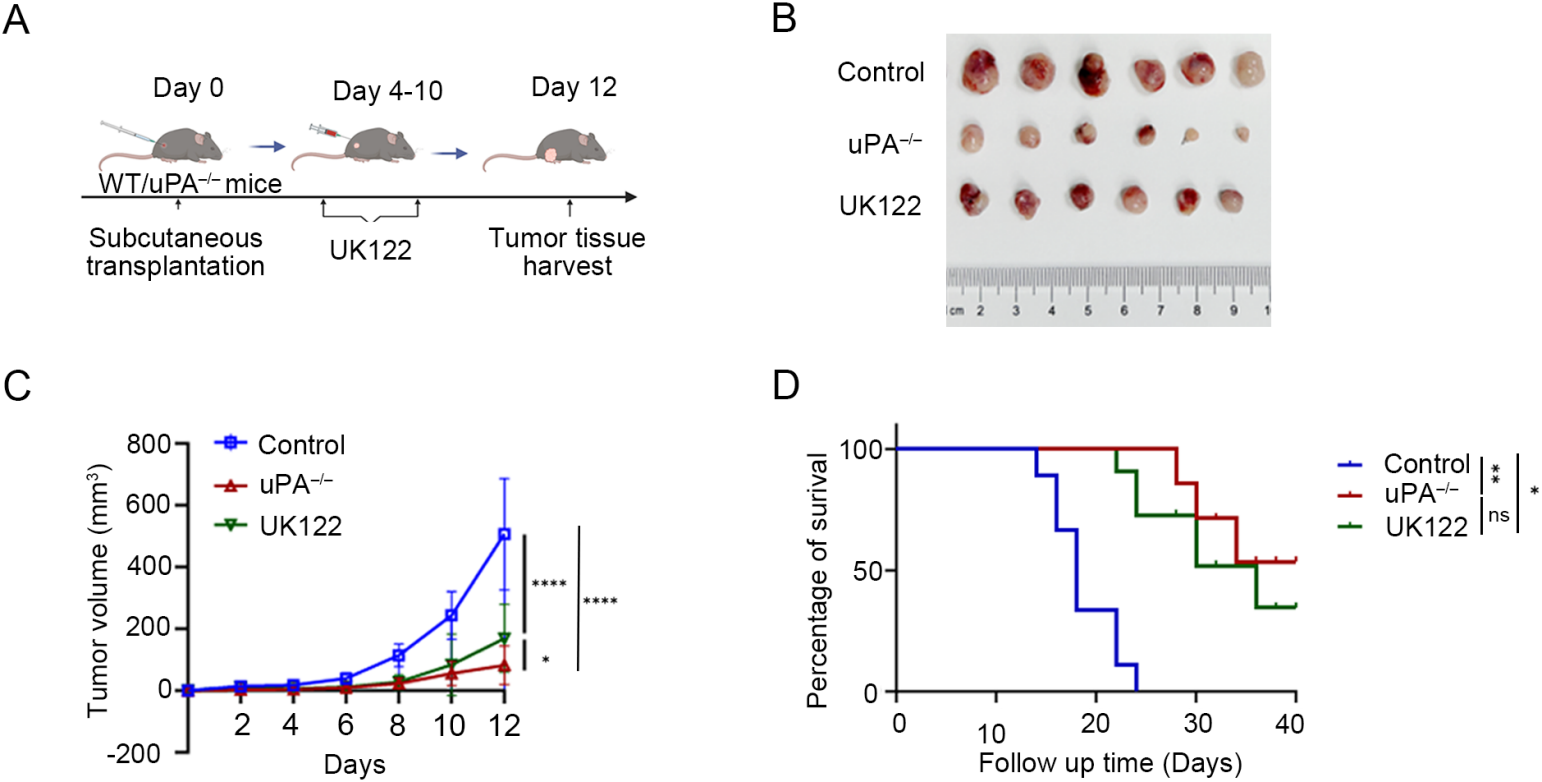
uPA deficiency inhibits prostate cancer progression. **(A)** Schematic diagram of RM-1 cells subcutaneous transplantation. **(B)** Subcutaneous tumors at the experimental endpoint were presented (each group, *n = 6*). **(C)** Tumor growth was determined by measuring the tumor volume every 2 days for 12 days. **(D)** Kaplan–Meier survival curves are shown (each group*, n = 6*). The results were presented as the mean ± SEM, ns, not statistically significant, * *P <* 0.05, ** *P <* 0.01, **** *P <* 0.0001.

To examine the functional role of uPA in prostate cancer development, we employed both genetic and pharmacological approaches. Using a uPA^–/–^ mouse model or administering the uPA inhibitor UK122 to WT mice, we found uPA deficiency significantly inhibited prostate cancer progression. Both models showed reduced tumor growth (WT: 492.64 ± 143.16 mm³; uPA^–/–^: 82.19 ± 59.01 mm³; UK122: 169.13 ± 104.96 mm³; **Figures 2A-C**) and prolonged survival (p<0.05; **Figure 2D**), with the genetic knockout exhibiting superior efficacy to pharmacological inhibition, collectively establishing that uPA deficiency inhibited prostate cancer progression.

### 3.3 uPA deficiency inhibits prostate cancer progression via activating antitumor immunity

**Figure 3:**
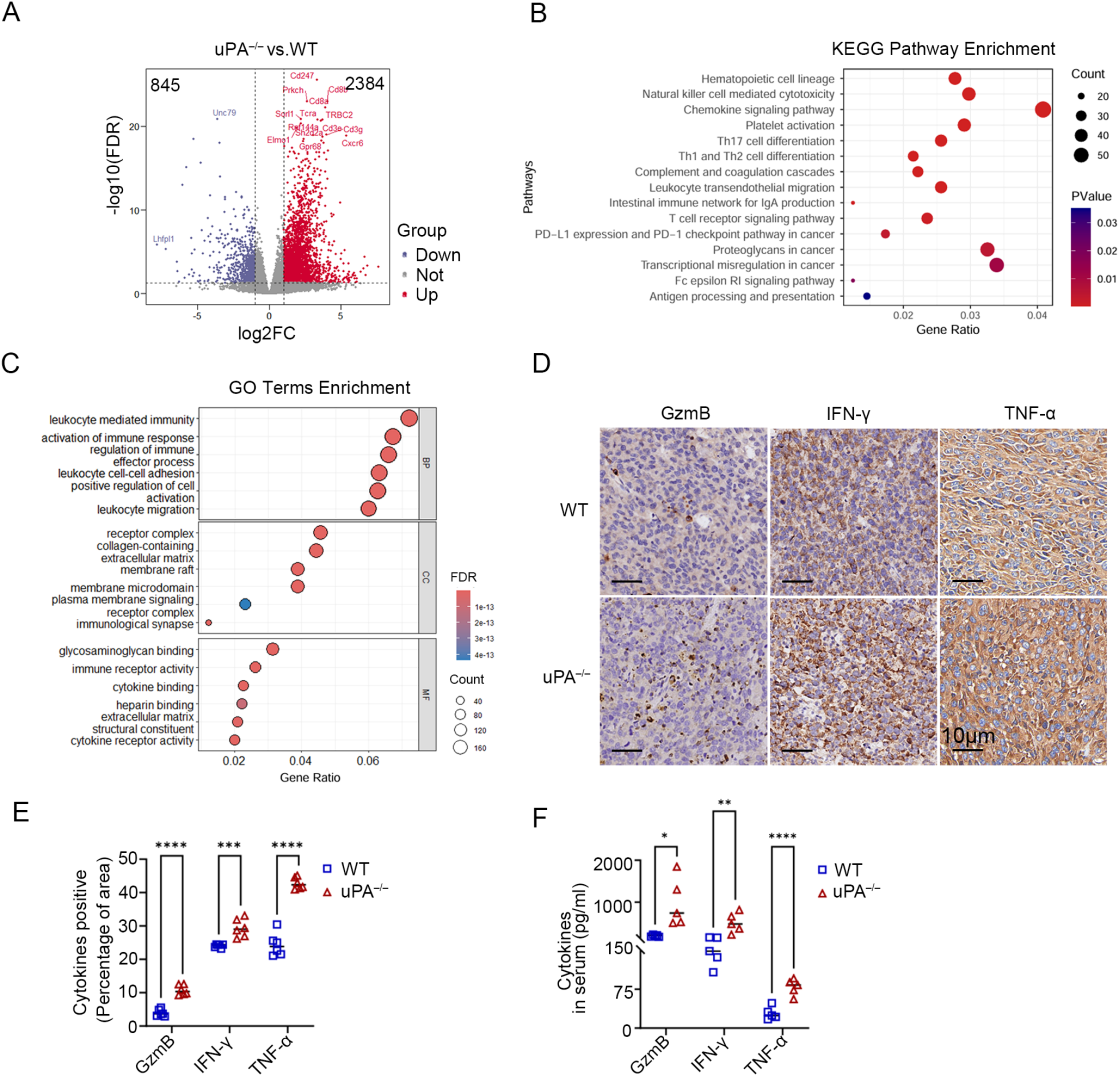
uPA deficiency inhibits prostate cancer progression via activating antitumor immunity. Volcano map **(A)**, KEGG pathway enrichment **(B)** and GO term enrichment analysis **(C)** of DEGs between the tumor tissues from WT and uPA^–/–^ tumor-bearing mice (each group, *n = 3*). **(D, E)** Immunostaining images and statistics of tumor sections from RM-1 tumor-bearing mice stained for GzmB, IFN-γ, and TNF-α (each group, *n = 6*); scale bar, 10 μm. **(F)** ELISA results showing the levels of secreted GzmB, IFN-γ, and TNF-α in the serum of WT and uPA^–/–^ mice 12 days after RM-1 cell injection (each group, *n = 5*). The results were presented as the mean ± SEM, * *P <* 0.05, ** *P <* 0.01, *** *P <* 0.001, **** *P <* 0.0001.

To characterize the alterations in the tumor microenvironment induced by uPA deficiency, RNA sequencing was used to identify 3,229 differentially expressed genes (DEGs) between the groups (2,384 upregulated and 845 downregulated in uPA^–/–^ mice; **Figure 3A**). KEGG pathway analysis revealed significant enrichment of immune-related pathways, including T cells receptor signaling, Th1/Th2/Th17 differentiation, chemokine signaling, and PD-1/PD-L1 checkpoint regulation (**Figure 3B**). GO term analysis further revealed the top-ranked biological processes, cellular components, and molecular functions associated with the DEGs (**Figure 3C**). Immunohistochemistry (IHC) confirmed elevated expression of the cytotoxic effector cytokines (GzmB, IFN-γ, TNF-α) in tumor tissues of uPA^–/–^ mice (**Figures 3D, E**), whereas ELISA confirmed increased serum levels of these cytokines in uPA^–/–^ tumor-bearing mice (**Figure 3F**). Together, these results suggested that uPA deficiency restrained prostate cancer progression via activating antitumor immunity.

### 3.4 uPA deficiency improves CD8^+^ T cells infiltration and cytotoxicity in prostate cancer

**Figure 4:**
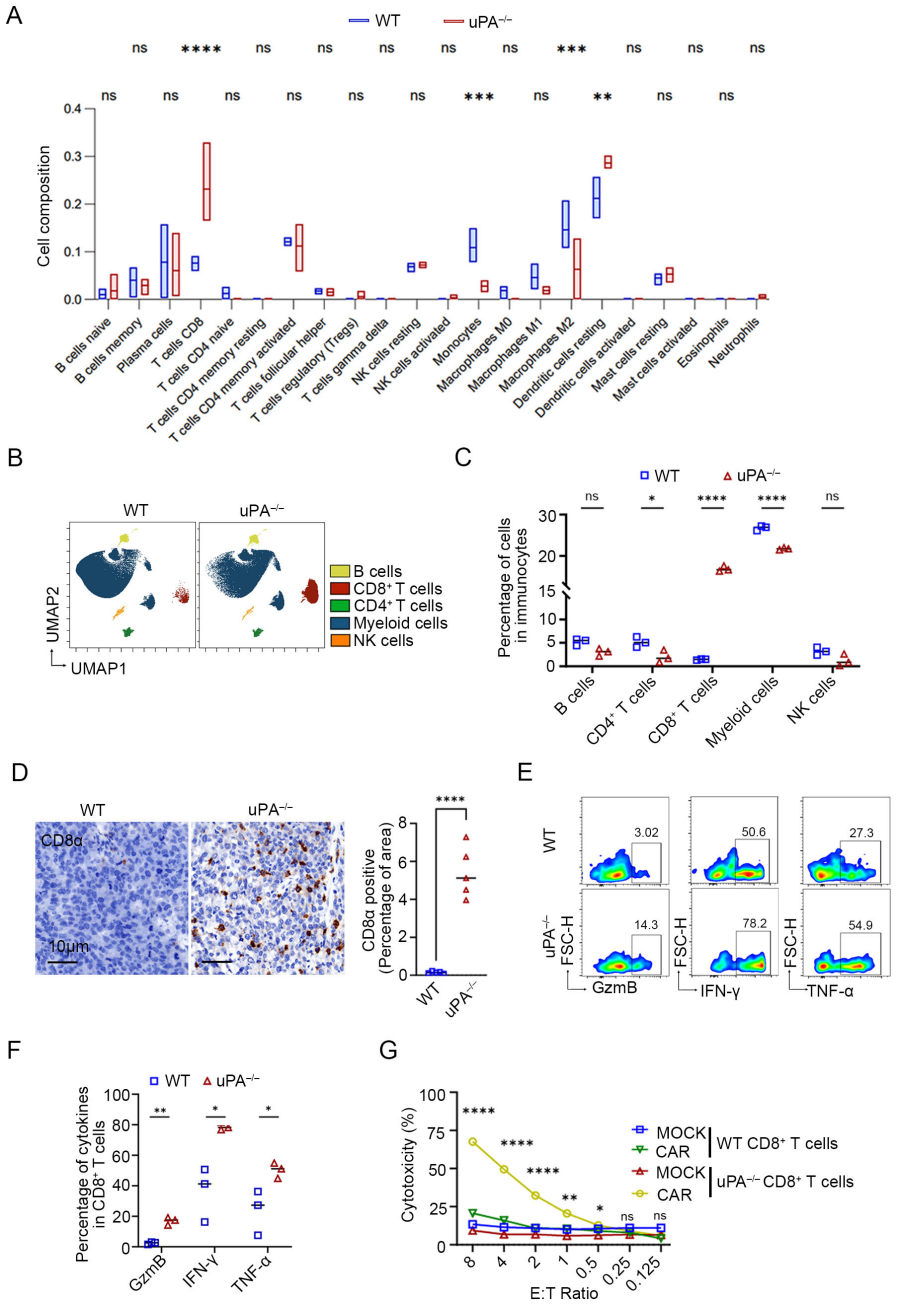
uPA deficiency improves CD8^+^ T cell infiltration and cytotoxicity in prostate cancer. **(A)** Box plots of the proportions of immune cells in tumors from the WT and uPA^–/–^ groups analyzed by the CIBERSORT algorithm (each group, *n = 3*). UMAP visualization **(B)** and quantitative analysis **(C)** of immune cell subsets, including B cells, CD4^+^ T cells, CD8^+^ T cells, myeloid cells, and NK cells (each group, *n=3*), as analyzed by CyTOF. **(D)** Immunostaining images of tumor sections from WT and uPA^–/–^ tumor-bearing mice stained for CD8α (each group, *n = 5*). Scale bar, 10 μm. Flow cytometry plots **(E)** and quantification **(F)** of GzmB, IFN-γ, and TNF-α in intratumoral CD8^+^ T cells from the WT and uPA^–/–^ mice (each group, *n = 3*). **(G)** LDH levels in the supernatants of cocultures of CD8^+^ CAR T cells or CD8^+^MOCK T cells (Effector, E) from the spleens of WT and uPA^–/–^ mice with RM1-CD19 cells (Target, T) at different E:T ratios after 24 h (each group, *n = 3*). The results were presented as the mean ± SEM, ns, not statistically significant, * *P <* 0.05, ** *P <* 0.01, *** *P <* 0.001, **** *P <* 0.0001.

Immune cell composition analysis using the CIBERSORT algorithm (34) demonstrated a marked increase in CD8^+^ T cells and resting dendritic cells, coupled with a significant reduction in myeloid populations (including monocytes and M2 macrophages), within the tumor tissues of uPA^–/–^ mice compared to WT mice (**Figure 4A**). CyTOF analysis of immune cell surface markers (**Supplementary Figure 1A**) further corroborated that CD8^+^ T cells were robustly elevated in tumors from uPA^–/–^ mice, in contrast with downregulated CD4^+^ T cells and myeloid subsets (**Figures 4B, C**). Flow cytometry and IHC further validated the accumulation of CD8^+^ T cells in tumors from uPA^–/–^ mice compared with WT mice (**Figure 4D**; **Supplementary Figures 1B, C**). These findings collectively suggested that the antitumor effects of uPA deficiency may be mediated through enhanced CD8^+^ T cells infiltration and functional activation.

Cytotoxic effector cytokines in intratumoral CD8^+^ T cells were detected by flow cytometry. Compared to WT group, intratumoral CD8^+^ T cells isolated from uPA^–/–^ mice exhibited significantly elevated secretion of GzmB, IFN-γ, TNF-α (**Figures 4E, F**), indicating that an enrichment of effector CD8^+^ T cells, a subset distinguished by acquiring an optimized effector differentiation profile, conferring potent antitumor cytotoxic activity (32). However, splenic uPA^–/–^ CD8^+^ T cells selectively upregulated IFN-γ production but showed no concurrent increase in GzmB or TNF-α levels (**Supplementary Figure 2**), suggesting that CD8^+^ T cells cytotoxicity in this context was tumor microenvironment-dependent. To evaluate the antitumor efficacy of uPA^–/–^ CD8^+^ T cells, we generated WT/uPA^–/–^ CD8^+^ CAR-T cells with anti-CD19 CAR, with unmodified counterparts serving as controls (WT/uPA^–/–^ CD8^+^ MOCK-T). Using RM1-CD19 cells (**Supplementary Figure 3**), uPA^–/–^ CD8^+^ CAR-T cells executed significantly stronger cytotoxicity than WT group at effector-to-target (E: T) ratios ≥0.5 (**Figure 4G**). Most strikingly, at the E:T ratio of 8:1, uPA^–/–^ CD8^+^ CAR-T cells exhibited 3.2-fold greater cytotoxicity (uPA^–/–^ CD8^+^ CAR-T cells: 67.66 ± 0.87%; WT CD8^+^ CAR-T cells: 20.70 ± 0.73%, p<0.0001). We further assessed the migratory capacity of splenic CD8^+^ T cells toward RM-1 cell supernatants. uPA^–/–^ CD8^+^ T cells displayed superior migration at ratios ≥0.5:1 (CD8^+^ T:RM-1) (**Supplementary Figure 4**). These findings demonstrated that uPA deficiency amplifies CD8^+^ T cells infiltration, cytotoxicity, and migratory capacity, positioning uPA^–/–^ CD8^+^ T cells as a superior candidate for adoptive cell therapy.

### 3.5 uPA deficiency inhibits prostate cancer progression in a manner dependent on CD8^+^ T cells.

**Figure 5:**
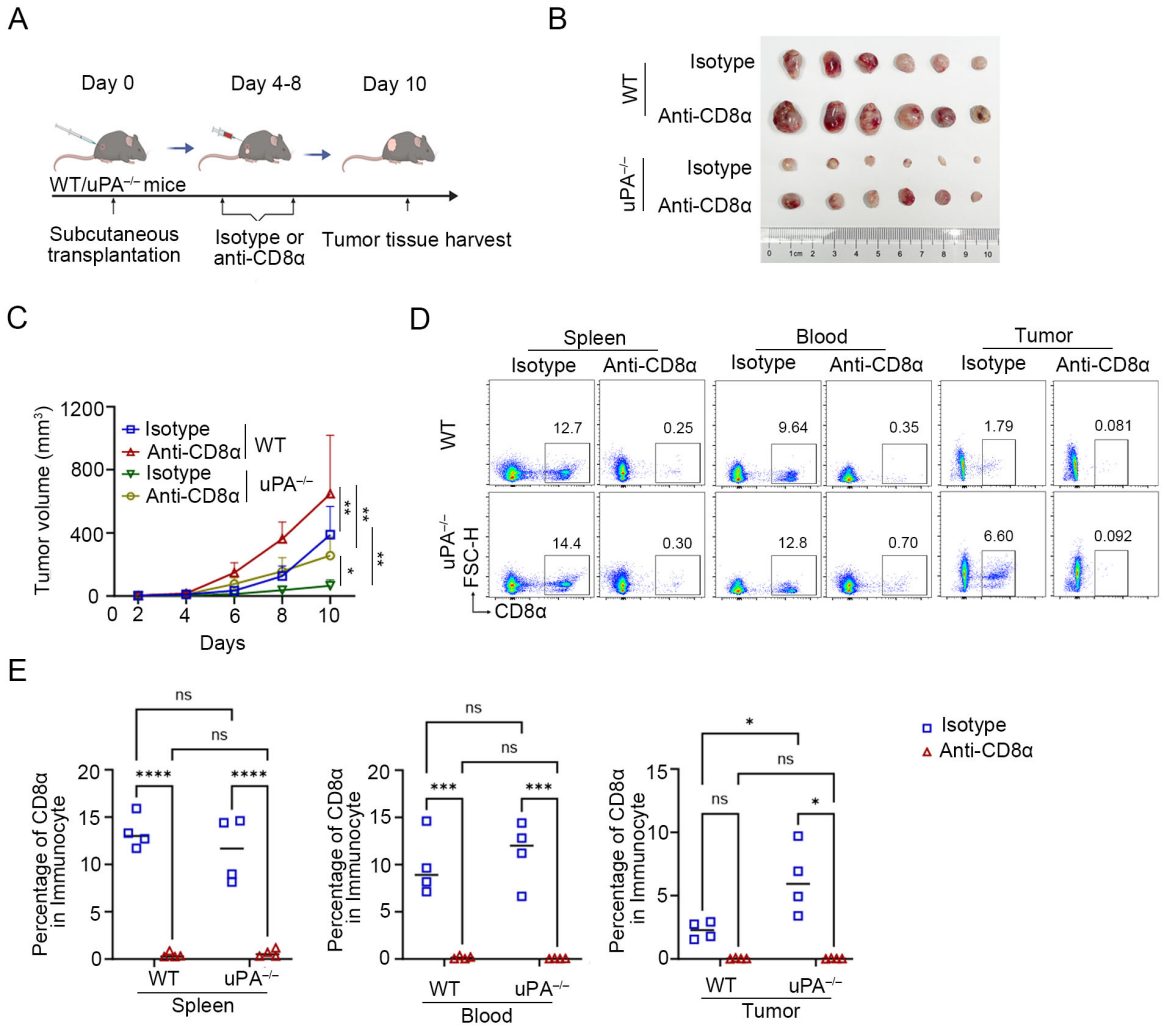
uPA deficiency inhibits prostate cancer progression in a manner dependent on CD8^+^ T cells. **(A)** Schematic diagram of the subcutaneous transplantation model. RM-1 cells were injected into the backs of WT (*n = 12*) and uPA^–/–^ (*n = 12*) mice. Anti-CD8α or isotype antibodies (100 μg/mouse) were injected into WT and uPA^–/–^ mice every 2 days starting on the 4^th^ day after subcutaneous transplantation. The tumor volume of the mice was consistently measured for up to 10 days (each group, *n = 6*). **(B)** Subcutaneous tumors in WT and uPA^–/–^ mice 12 days after RM-1 cell injection (each group, *n =6*). **(C)** Tumor growth curves with mean tumor volumes ± SEM (each group, *n = 6*). Flow cytometry plots **(D)** and statistics **(E)** of the population of CD8^+^ T cell in the blood, spleen, and tumors in different groups (each group, *n =4*). The results were presented as the mean ± SEM, ns, not statistically significant, *P < 0.05, **P < 0.01, ***P < 0.001, ****P < 0.0001.

We administered anti-CD8α or isotype antibodies to WT and uPA^–/–^ tumor-bearing mice (**Figure 5A**). Compared with isotype control group, depletion of CD8^+^ T cells with anti-CD8α antibodies accelerated tumor progression in both WT and uPA^–/–^ mice (WT- Isotype: 388.70 ± 163.53 mm^3^; WT- Anti-CD8α: 659.83 ± 329.32 mm^3^; uPA^–/–^ Isotype: 65.39 ± 34.54 mm^3^; uPA^–/–^ Anti-CD8α: 256.24 ± 109.25 mm^3^; **Figures 5B, C**), which underscored the centrality of CD8^+^ T cells in antitumor immunity. Although CD8^+^ T cells depletion partially reversed tumor suppression in uPA^–/–^ mice, the resultant tumor volumes remained 21% smaller than WT isotype controls (**Figure 5B**). This result demonstrated that uPA deficiency mediated tumor suppression primarily through CD8^+^ T cells-dependent mechanisms, while potentially involving CD8^+^ T cells-independent pathways. Flow cytometry confirmed near-complete elimination of CD8^+^ T cells in the peripheral blood, spleen, and tumors across both groups (**Figures 5D, E**), which validated the specificity and efficacy of the depletion regimen.

### 3.6 uPA deficiency enhances the expression of PD-1 on intratumoral CD8^+^ T cells

**Figure 6:**
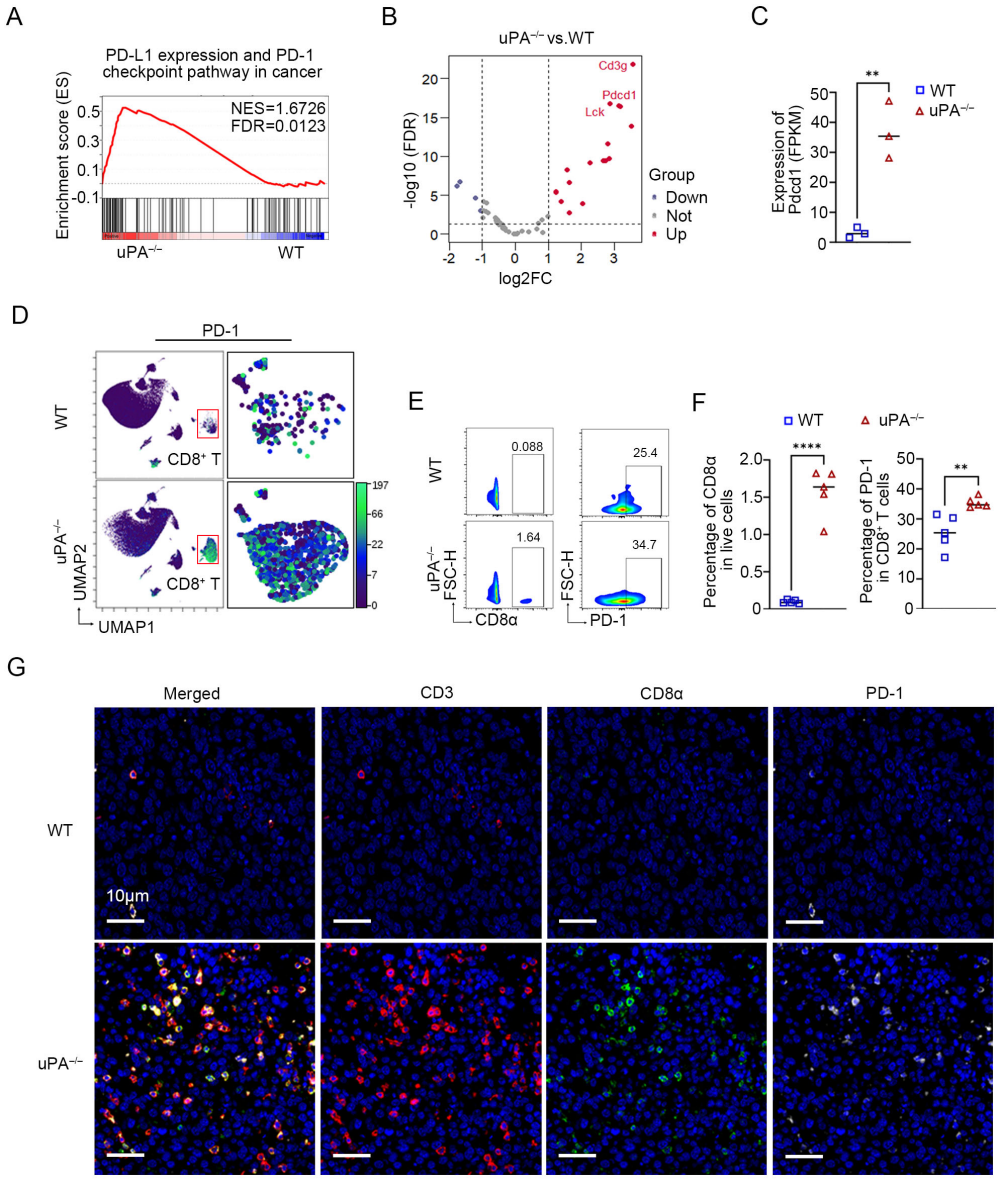
uPA deficiency enhances the expression of PD-1 on intratumoral CD8^+^ T cells. **(A)** GSEA showing PD-L1 expression and the PD-1 checkpoint pathway in cancer pathways enriched in the uPA^–/–^ group. **(B)** Volcano map of DEGs associated with PD-L1 expression and the PD-1 (pdcd1) checkpoint pathway between groups (each group, *n = 3*). **(C)** Blotting of the FPKM value of PD-1 (pdcd1) between groups. **(D)** UMAP visualization of the expression of PD-1 (pdcd1) in CD8^+^ T cells detected by CyTOF. Flow cytometry plots **(E)** and statistics **(F)** of the populations of intratumoral PD-1^+^CD8^+^ T cells in different groups (each group, *n =5*). **(G)** mIHC was used to detect the expression of PD-1 in intratumoral CD8^+^ T cells. Scale bar, 10 μm. The results were presented as the mean ± SEM, ** *P <* 0.01, **** *P <* 0.0001.

The PD-1/PD-L1 axis is a central regulator of T and B cell activation, maintaining immune tolerance through inhibitory signaling (33). In solid tumors, the upregulation of PD-L1 on malignant cells engages PD-1 receptors on T cells, thereby dampening cytotoxic activity and facilitating immune evasion (37, 38). RNA sequencing revealed profound PD-1 upregulation in tumors from uPA^–/–^ mice compared to WT mice (**Figures 6A–C**). This finding was corroborated by CyTOF and flow cytometry, which demonstrated elevated intratumoral PD-1^+^ CD8^+^ T cells infiltration in uPA^–/–^ mice (**Figures 6D-F**). mIHC further demonstrated that more PD-1^+^CD8^+^ T cells in tumor tissues from uPA^–/–^ mice (**Figure 6G**). Collectively, uPA deficiency drove a higher frequency of exhaustion-prone CD8^+^ T cells, characterized by elevated PD-1 expression.

### 3.7 uPA inhibitor enhances the efficacy of anti-PD-1 therapy in prostate cancer

**Figure 7:**
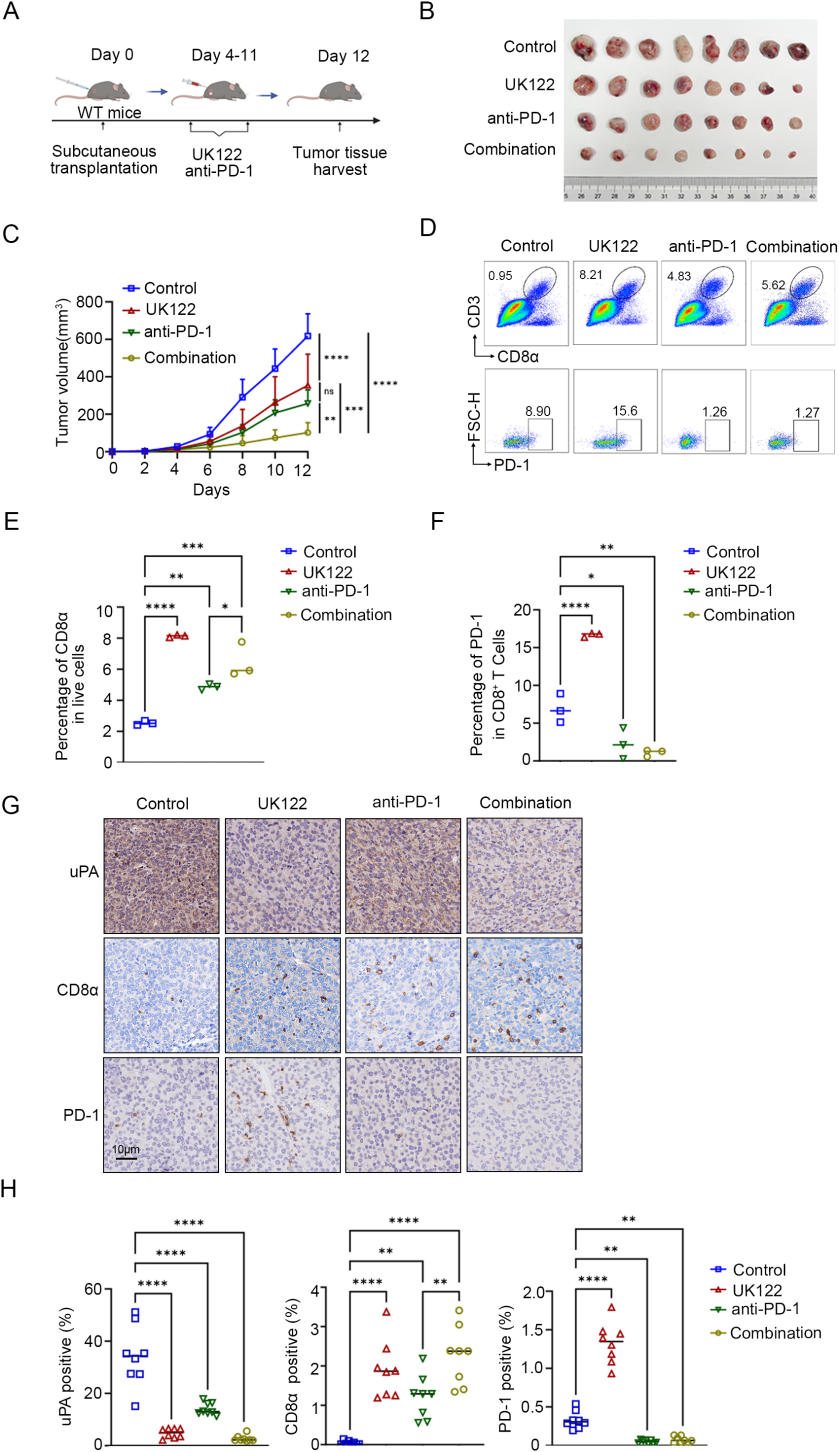
uPA inhibitor enhances the efficacy of anti-PD-1 therapy in prostate cancer. **(A)** Schematic diagram of the subcutaneous tumor model in WT mice. RM-1 cells were subcutaneously injected into WT mice (*n = 32*). Four days after RM-1 cells injection, the mice were randomized into four groups: (i) the control group (100 μl of DMSO, *n = 8*); (ii) the UK122 group (4 mg/kg, *n = 8*); (iii) the anti-PD-1 group (100 μg/mouse, *n = 8*); and (iv) the combination group (4 mg/kg UK122 and 100 μg/mouse anti-PD-1),. **(B)** Subcutaneous tumors formed from RM-1 cells in WT and uPA^–/–^ mice after 12 days of treatment (each group*, n = 8*). **(C)** Tumor growth curves with the mean tumor volume ± SEM (each group*, n = 8*). Flow cytometry plots **(D)** and statistics **(E, F)** of the percentage of intratumoral CD8^+^ T cells and the expression of PD-1 in CD8^+^ T cells (each group, *n = 3*). Immunostaining images **(G)** and statistics **(H)** of tumor sections from RM-1 tumor-bearing mice stained for uPA, CD8a, and PD-1 (each group, *n = 8*); scale bar, 10 μm. The results were presented as the mean ± SEM, ns, not statistically significant, * *P <* 0.05, ** *P <* 0.01, *** *P <* 0.001, **** *P <* 0.0001.

To evaluate the synergistic potential of uPA inhibitor and immune checkpoint blockade, we treated WT mice with (1) DMSO, (2) UK122 monotherapy (4 mg/kg), (3) anti-PD-1 monotherapy (100 μg), or (4) the combination of UK122 and anti-PD-1 (**Figure 7A**). Both monotherapies suppressed tumor growth, yet the combination regimen achieved superior efficacy, suggesting additive antitumor effects (Control: 617.93 ± 118.01 mm^3^; UK122: 257.78 ± 71.54 mm^3^; anti-PD-1: 353.77 ± 167.47 mm^3^; combination: 100.16 ± 49.79 mm^3^, **Figures 7B, C**). Flow cytometry and immunostaining revealed more CD8^+^ T cells infiltration in tumors treated with UK122 or anti-PD-1 monotherapy, and combination therapy. Notably, although combination therapy enhanced CD8^+^ T cells infiltration compared to anti-PD-1 monotherapy, it did not exceed UK122 monotherapy levels, suggesting that the superior efficacy likely stemmed from the concerted actions of UK122 promoting T cells recruitment and anti-PD-1 restoring cellular functionality (**Figures 7D-H**).

## 4 Discussion

This study established uPA as an orchestrator of immunosuppression in prostate cancer. Analysis of human specimens revealed a significant inverse correlation between uPA expression and intratumoral CD8^+^ T cells density, positioning uPA as a therapeutic target. Previous study reported that uPA deficiency suppresses tumor growth and reduced macrophage infiltration in murine models (28), we further investigated the multifaceted impact of uPA on the prostate tumor microenvironment, with a focus on the interactions of tumor and immune cell. Integrated CIBERSORT and CyTOF profiling demonstrated that genetic uPA deficiency simultaneously enhanced CD8^+^ T cells infiltration and decreased immunosuppressive myeloid populations within prostate cancer. Tumor-associated macrophages restrict the function of CD8^+^ T cells, which in turn constrain myeloid cell accumulation in pre-metastatic tissue (34, 35). However, the mechanistic link between the enrichment of uPA^–/–^ CD8^+^ T cells and myeloid cell depletion remains to be investigated.

Our *in vitro* studies revealed that uPA^–/–^ CD8^+^ T cells exhibited enhanced tumor-killing activity through the secretion of GzmB, IFN-γ, and TNF-α. CAR T cells provides a powerful experimental platform to investigate the tumor-specific cytotoxicity of T cells (36). Anti-CD19 CAR-T cells are genetically engineered to express synthetic receptors that directly target CD19, a tumor-associated surface antigen, independent of MHC presentation (37). This allowed for precise evaluation of uPA^–/–^ CD8^+^ T cells functionality. By introducing anti-CD19 CAR into WT and uPA^–/–^CD8^+^ T cells, we found that uPA^–/–^CD8^+^ T cells exhibited increased cytotoxicity. Strikingly, when CD8^+^ T cells was depleted in uPA^–/–^ mice, the tumors resumed growth but remained significantly smaller than those in WT-Isotype control group, suggesting that uPA deficiency may exert additional tumor-suppressive effects, possibly through direct modulation of tumor cells or other immune cell populations. Therefore, we recommend a therapeutic approach that involves knockout of uPA in CD8^+^ T cells and subsequent reinfusion into patients. This strategy is designed to increase the tumor infiltration and cytotoxic activity of CD8^+^ T cells to facilitate tumor elimination.

Our findings demonstrated that uPA deficiency or pharmacological inhibition enhanced intratumoral CD8^+^ T cells infiltration and cytotoxicity; these cells partly exhibited PD-1 expression during tumor progression. While initial PD-1 upregulation indicates T cells activation, sustained PD-1/PD-L1 signaling promotes T cells exhaustion (34), explaining the limited tumor clearance despite robust CD8^+^ T cells expansion. The enhanced therapeutic efficacy of combined uPA inhibition and anti-PD-1 therapy resulted from PD-1 blockade-mediated reversal of CD8^+^ T cells exhaustion and restoration of sustained antitumor immunity (35, 36). Collectively, these results position uPA inhibition as a promising strategy to potentiate antitumor immunity and improve immunotherapy outcomes in prostate cancer, with the combination regimen demonstrating significantly enhanced efficacy compared to monotherapies. Yet the regulatory mechanisms of uPA in PD-1 upregulation and immune checkpoint regulation remain to be further elucidated, for instance, by using CRISPR-based screening coupled with transcriptomic analysis in uPA^–/–^CD8^+^ T cells.

**Figure 8:**
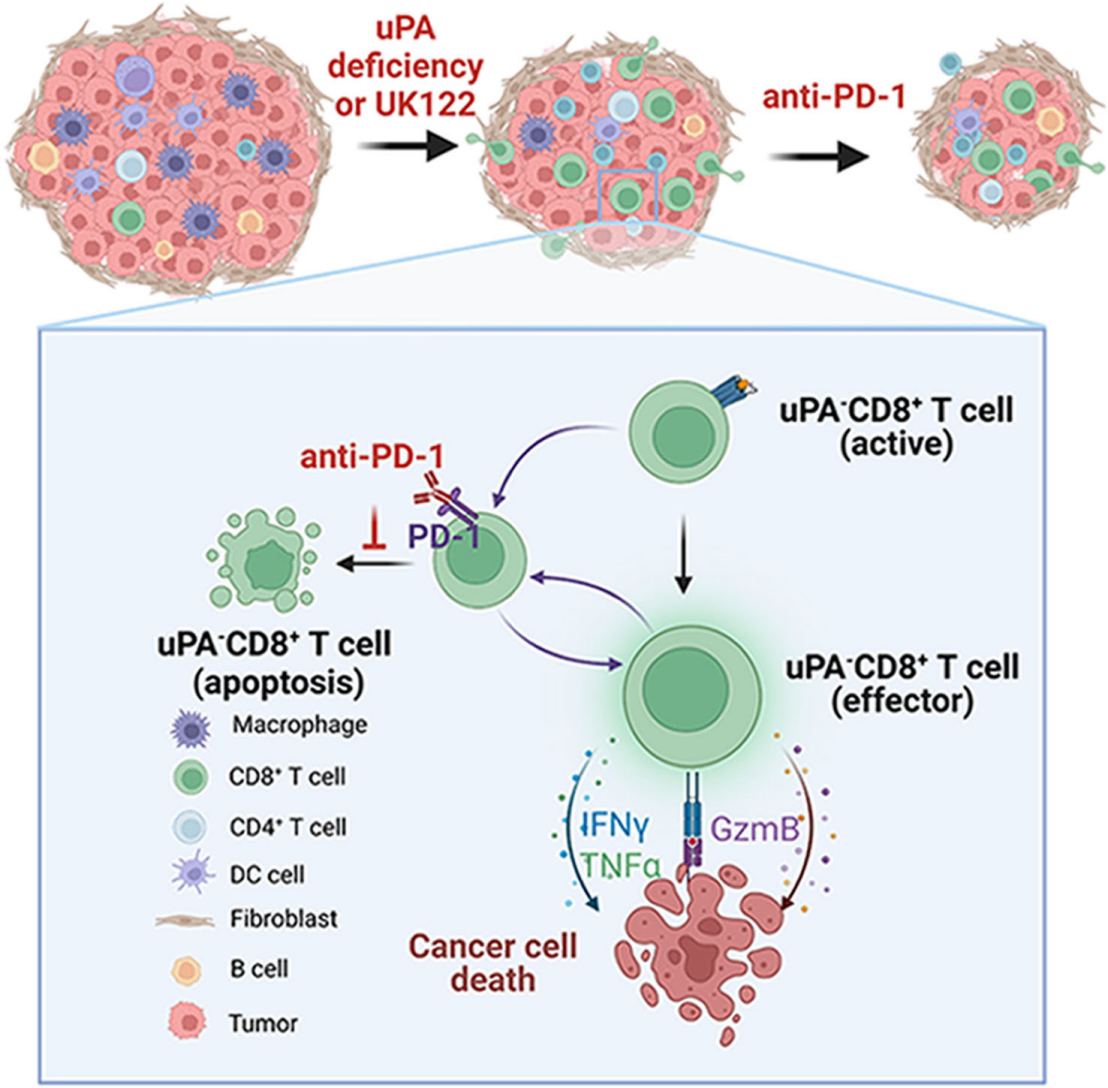
Graphical schematic showing the role of uPA deficiency in CD8^+^ T-cell-mediated antitumor immunity. uPA deficiency results in increased CD8^+^ T-cell infiltration and cytotoxicity via increased GzmB, IFN-γ, and TNF-α secretion, further suppressing tumor growth. In addition, uPA deficiency or downregulation increases the percentage of PD-1-expressing CD8^+^ T-cells, which enhances sensitivity to anti-PD-1 therapy. Together, these findings indicate that uPA can potentially become an effective target for enhancing tumor immunity.

In summary, we proposed the hypothetical model illustrated in **Figure 8**. uPA deficiency resulted in increased CD8^+^ T-cells infiltration and cytotoxicity via increasing GzmB, IFN-γ, and TNF-α secretion, further suppressing tumor growth. In addition, uPA deficiency or downregulation increased the percentage of PD-1^+^CD8^+^ T cells, which enhanced sensitivity to anti-PD-1 therapy. Together, these findings indicated that uPA can potentially become an effective target for enhancing tumor immunity.

## Data Availability

The datasets presented in this study can be found in online repositories. The names of the repository/repositories and accession number(s) can be found below: https://www.ncbi.nlm.nih.gov/, PRJNA1251269.

## Supplementary material

Supplementary Figure 1 uPA deficiency improves immunocyte infiltration. **(A)** UMAP plots showed the cell cluster markers detected by CyTOF (*n = 3*). **(B, C)** Representative images and statistics of intratumoral immunocytes, myeloid cells, B cells, T cells, CD8^+^ T cells, and CD4^+^ T cells between the WT and uPA^–/–^ groups (*n = 3*).

Supplementary Figure 2 uPA deficiency increases the expression of IFN-γ in splenic CD8^+^ T cells. **(A)** Representative flow cytometry plots showing the expression levels of cytotoxic molecules of T cells, such as TNF-α, IFN-γ, and GzmB, in Control and uPA^–/–^ CD8^+^ T cells of the spleen (*n = 3*). **(B)** Percentage of GzmB, IFN-γ, and TNF-α in Control or uPA^–/–^ CD8^+^ T cells (*n = 3*).

Supplementary Figure 3 Establishment and validation of WT CD8^+^CAR-T cells, uPA^–/–^ CD8^+^CAR-T cells, and RM1-hCD19. **(A)** Representative flow cytometry plots showing the expression levels of CD19 CAR in WT and uPA^–/–^ CD8^+^ CAR-T T cells. **(B)** Representative flow cytometry plots showing the expression levels of CD19 in RM-1 cells.

Supplementary Figure 4 uPA deficiency increases migrations of CD8^+^ T cells at different ratios. **(A–C)** Schematic diagram, flow cytometry plots, and statistics of migratory CD8^+^ T cells recruited by RM-1 cells after co-culturing at different ratios for 24 h (*n = 3*).

## References

[1] Li Y Zhu K Wang L Zhang Y Hou S Wang W. Effectiveness of web-based intervention on reducing symptom burden, improving self-management capabilities and self-efficacy among prostate cancer survivors: A systematic review and meta-analysis protocol. BMJ Open. (2024) 14:e082709. doi: 10.1136/bmjopen-2023-082709, PMID: 38821569 PMC11149145

[2] Jiang J Chen B Tang B Yang J Zhang T LI J. Trends of prostate cancer morbidity in low-incidence countries from 1990–2019. Cancer Epidemiol Biomarkers Prev. (2024) 33:186–95. doi: 10.1158/1055-9965.EPI-23-1034, PMID: 38317630 PMC10844848

[3] Dai B Wang H Shi B Xing J Zhu S He Z. CACA guidelines for holistic integrative management of prostate cancer. Holistic Integr Oncol. (2024) 3:47. doi: 10.1007/s44178-024-00118-4, PMID: 37520336 PMC9255514

[4] Siegel RL Kratzer TB Giaquinto AN Sung H Jemal A. Cancer statistics. CA Cancer J Clin. (2025) 75(1):10–45. doi: doi: 10.3322/caac.21871, PMID: 39817679 PMC11745215

[5] Kwan EM Spain L Anton A Gan CL Garrett L Chang D. Avelumab combined with stereotactic ablative body radiotherapy in metastatic castration-resistant prostate cancer: the phase 2 ICE-PAC clinical trial. Eur Urol. (2022) 81:253–62. doi: 10.1016/j.eururo.2021.08.011, PMID: 34493414

[6] Wang DR Wu XL Sun YL. Therapeutic targets and biomarkers of tumor immunotherapy: response versus non-response. Signal Transduct Target Ther. (2022) 7:331. doi: 10.1038/s41392-022-01136-2, PMID: 36123348 PMC9485144

[7] Sharma P Pachynski RK Narayan V Fléchon A Gravis G Galsky MD. Nivolumab plus ipilimumab for metastatic castration-resistant prostate cancer: preliminary analysis of patients in the checkMate 650 trial. Cancer Cell. (2020) 38:489–499.e3. doi: 10.1016/j.ccell.2020.08.007, PMID: 32916128

[8] Powles T Yuen KC Gillessen S Kadel EE Rathkopf D Matsubara N. Atezolizumab with enzalutamide versus enzalutamide alone in metastatic castration-resistant prostate cancer: a randomized phase 3 trial. Nat Med. (2022) 28:144–53. doi: 10.1038/s41591-021-01600-6, PMID: 35013615 PMC9406237

[9] Janiczek M Szylberg Ł Kasperska A Kowalewski A Parol M Antosik P. Immunotherapy as a promising treatment for prostate cancer: A systematic review. J Immunol Research 2017. (2017) p:1–6. doi: 10.1155/2017/4861570, PMID: 29109964 PMC5646317

[10] Rehman LU Nisar MH Fatima W Sarfraz A Azeem N Sarfraz Z. Immunotherapy for prostate cancer: A current systematic review and patient centric perspectives. J Clin Med. (2023) 12:1446. doi: 10.3390/jcm12041446, PMID: 36835981 PMC9966657

[11] Olson BM Jankowska-Gan E Becker JT Vignali DA Burlingham WJ. Human prostate tumor antigen–specific CD8+ Regulatory T cells are inhibited by CTLA-4 or IL-35 blockade. J Immunol. (2012) 189:5590–601. doi: 10.4049/jimmunol.1201744, PMID: 23152566 PMC3735346

[12] Peranzoni E Lemoine J Vimeux L Feuillet V Barrin S Kantari-Mimoun C. Macrophages impede CD8 T cells from reaching tumor cells and limit the efficacy of anti–PD-1 treatment. Proc Natl Acad Sci. (2018) 115(17):E4041–50. doi: 10.1073/pnas.1720948115, PMID: 29632196 PMC5924916

[13] Yang Y Attwood K Bshara W Mohler JL Guru K Xu B. High intratumoral CD8+ T-cell infiltration is associated with improved survival in prostate cancer patients undergoing radical prostatectomy. Prostate. (2020) 81:20–8. doi: doi: 10.1002/pros.24068, PMID: 33085799 PMC9869431

[14] Mo RJ Han ZD Liang YK Ye JH Wu SL Lin SX. Expression of PD-L1 in tumor-associated nerves correlates with reduced CD8+ Tumor-associated lymphocytes and poor prognosis in prostate cancer. Int J Cancer. (2019) 144:3099–110. doi: 10.1002/ijc.32061, PMID: 30537104

[15] Jung J Park SY Park JY Kim D Lee K Choi S. Reactivation of varicella-zoster virus in patients with lung cancer receiving immune checkpoint inhibitors: retrospective nationwide population- based cohort study of the health insurance review and assessment database in South Korea. Cancers (Basel). (2024) 16(8):1499. doi: 10.20944/preprints202402.0449.v1 38672581 PMC11048333

[16] Maute RL Gordon SR Mayer AT McCracken MN Natarajan A Ring NG. Engineering high-affinity PD-1 variants for optimized immunotherapy and immuno-pet imaging. Proc Natl Acad Sci. (2015) 112(47):E6506–14. doi: 10.1073/pnas.1519623112, PMID: 26604307 PMC4664306

[17] Shi Z Du Q Wang X Wang J Chen H Lang Y. Granzyme B in circulating CD8+ T cells as a biomarker of immunotherapy effectiveness and disability in neuromyelitis optica spectrum disorders. Front Immunol. (2022) 13:1027158. doi: 10.3389/fimmu.2022.1027158, PMID: 36439094 PMC9682179

[18] St Paul M Ohashi PS. The roles of CD8(+) T cell subsets in antitumor immunity. Trends Cell Biol. (2020) 30:695–704. doi: doi:10.1016/j.tcb.2020.06.003, PMID: 32624246

[19] Redman J Gulley JL Madan RA. Combining immunotherapies for the treatment of prostate cancer. Urologic Oncol Semin Original Investigations. (2017) 35:694–700. doi: 10.1016/j.urolonc.2017.09.024, PMID: 29146441 PMC6599516

[20] Han KY Chen PN Hong MC Hseu YC Chen KM Hsu LS. Naringenin Attenuated Prostate Cancer Invasion via Reversal of Epithelial–to–Mesenchymal Transition and Inhibited uPA Activity. Anticancer Res. (2018) 38:6753–8. doi: 10.21873/anticanres.13045, PMID: 30504386

[21] Semina EV Rubina KA Shmakova AA Rysenkova KD Klimovich PS Aleksanrushkina NA. Downregulation of uPAR promotes urokinase translocation into the nucleus and epithelial to mesenchymal transition in neuroblastoma. J Cell Physiol. (2020) 235:6268–86. doi: 10.1002/jcp.29555, PMID: 31990070 PMC7318179

[22] Smith HW Marshall CJ. Regulation of cell signalling by uPAR. Nat Rev Mol Cell Biol. (2010) 11:23–36. doi: 10.1038/nrm2821, PMID: 20027185

[23] Zhu M Gokhale VM Szabo L Munoz RM Baek H Bashyam S. Identification of a novel inhibitor of urokinase-type plasminogen activator. Mol Cancer Ther. (2007) 6:1348–56. doi: 10.1158/1535-7163.MCT-06-0520, PMID: 17431113

[24] Khatib AM Nip J Fallavollita L Lehmann M Jensen G Brodt P. Regulation of urokinase plasminogen activator/plasmin-mediated invasion of melanoma cells by the integr*in vitro*nectin receptor alphaVbeta3. Int J Cancer. (2001) 91:300–8. doi: 10.1002/1097-0215(200002)9999:9999<::AID-IJC1055>3.0.CO;2-N, PMID: 11169951

[25] Vassalli Jd Fau - Belin D Belin D. Amiloride selectively inhibits the urokinase-type plasminogen activator. FEBS Lett. (1987) 214(1):187–91. doi: 10.1016/0014-5793(87)80039-x, PMID: 3106085

[26] Chen J López-Moyado IF Seo H Lio CJ Hempleman LJ Sekiya T. NR4A transcription factors limit CAR T cell function in solid tumours. Nature. (2019) 567:530–4. doi: 10.1038/s41586-019-0985-x, PMID: 30814732 PMC6546093

[27] Halim L Das KK Larcombe-Young D Ajina A Candelli A Benjamin R. Engineering of an avidity-optimized CD19-specific parallel chimeric antigen receptor that delivers dual CD28 and 4-1BB co-stimulation. Front Immunol. (2022) 13:836549. doi: 10.3389/fimmu.2022.836549, PMID: 35222427 PMC8863855

[28] Camorani S Passariello M Agnello L Esposito S Collina F Cantile M. Aptamer targeted therapy potentiates immune checkpoint blockade in triple-negative breast cancer. J Exp Clin Cancer Res. (2020) 39:180. doi: 10.1186/s13046-020-01694-9, PMID: 32892748 PMC7487859

[29] Yoshida T Mihara K Takei Y Yanagihara K Kubo T Bhattacharyya J. All-trans retinoic acid enhances cytotoxic effect of T cells with an anti-CD38 chimeric antigen receptor in acute myeloid leukemia. Clin Transl Immunol. (2016) 5:e116. doi: 10.1038/cti.2016.73, PMID: 28090317 PMC5192064

[30] Lu LL Xiao SX Lin ZY Bai JJ Li W Song ZQ. GPC3-IL7-CCL19-CAR-T primes immune microenvironment reconstitution for hepatocellular carcinoma therapy. Cell Biol Toxicol. (2023) 39:3101–19. doi: 10.1007/s10565-023-09821-w, PMID: 37853185

[31] Evrard M Kwok IWH Chong SZ Teng KWW Becht E Chen J. Developmental analysis of bone marrow neutrophils reveals populations specialized in expansion, trafficking, and effector functions. Immunity. (2018) 48:364–379.e8. doi: 10.1016/j.immuni.2018.02.002, PMID: 29466759

[32] Niederlova V Tsyklauri O Kovar M Stepanek O. IL-2-driven CD8(+) T cell phenotypes: implications for immunotherapy.Trends Immunol. (2023) 44(11):890–901. doi: 10.1016/j.it.2023.09.003, PMID: 37827864 PMC7615502

[33] Sun C Mezzadra R Schumacher TN. Regulation and function of the PD-L1 checkpoint. Immunity. (2018) 48:434–52. doi: 10.1016/j.immuni.2018.03.014, PMID: 29562194 PMC7116507

[34] Zhang W Zhang C Li W Deng J Herrmann A Priceman SJ. CD8+ T-cell immunosurveillance constrains lymphoid premetastatic myeloid cell accumulation. Eur J Immunol. (2015) 45:71–81. doi: 10.1002/eji.201444467, PMID: 25310972 PMC4293284

[35] Tharp KM Kersten K Maller O Timblin GA Stashko C Canale FP. Tumor-associated macrophages restrict CD8+ T cell function through collagen deposition and metabolic reprogramming of the breast cancer microenvironment. Nat Cancer. (2024) 5:1045–62. doi: 10.1038/s43018-024-00775-4, PMID: 38831058 PMC12204312

[36] He J Xiong X Yang H Li D Liu X Li S. Defined tumor antigen-specific T cells potentiate personalized TCR-T cell therapy and prediction of immunotherapy response. Cell Res. (2022) 32:530–42. doi: 10.1038/s41422-022-00627-9, PMID: 35165422 PMC9160085

[37] Harris DT Kranz DM. Adoptive T cell therapies: A comparison of T cell receptors and chimeric antigen receptors. Trends Pharmacol Sci. (2016) 37:220–30. doi: 10.1016/j.tips.2015.11.004, PMID: 26705086 PMC4764454

