# Supplementary figures and images for: Urokinase-type plasminogen activator deficiency enhances CD8+ T cell infiltration and anti-PD-1 therapy efficacy in prostate cancer
Source: Front Immunol. 2025 Sep 1;16:1625226. doi: 10.3389/fimmu.2025.1625226 (PMC12434036; doi:10.3389/fimmu.2025.1625226)

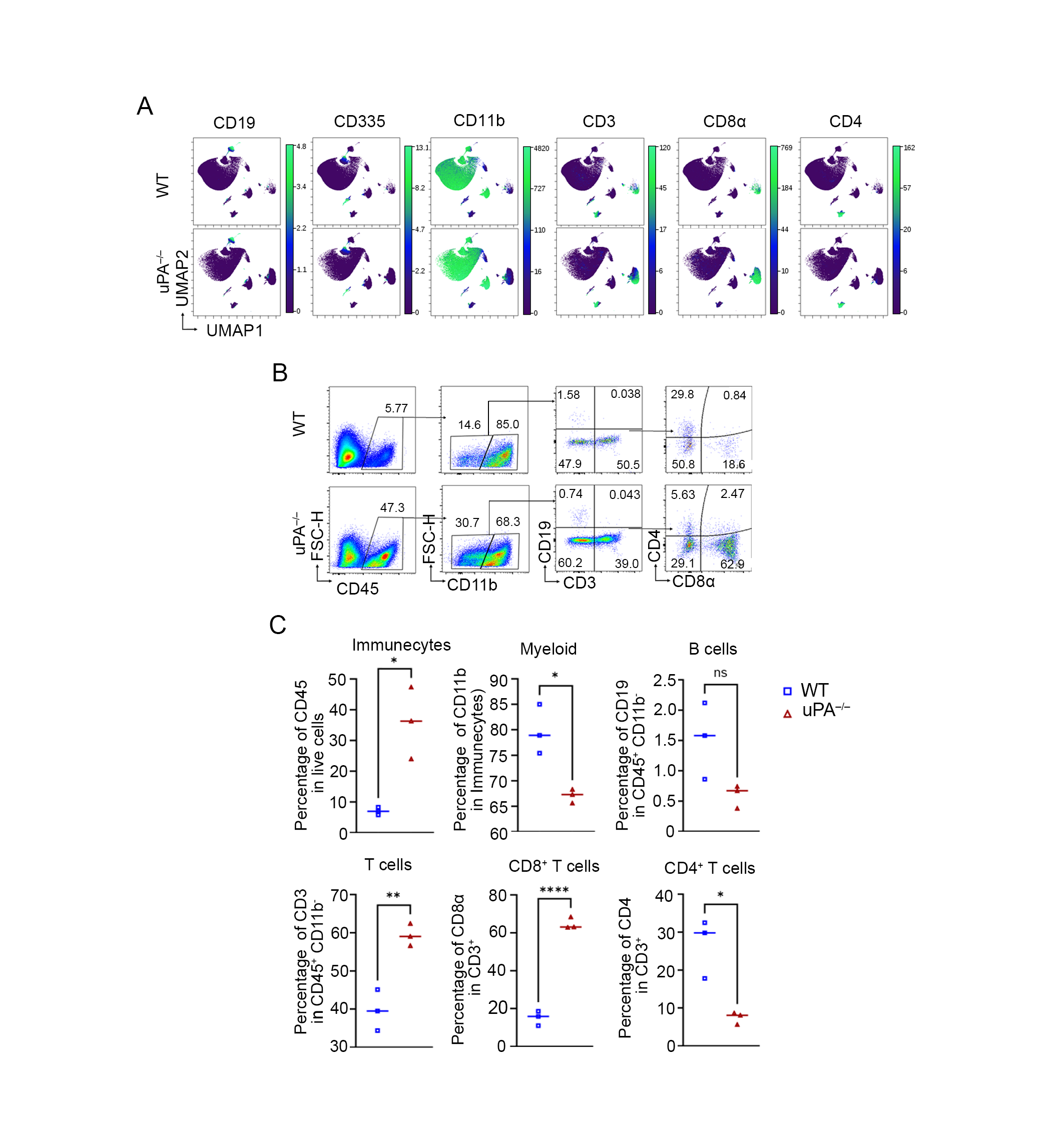

Supplement: Supplementary Figure 1 — uPA deficiency improves immunocyte infiltration. (A) UMAP plots showed the cell cluster markers detected by CyTOF (n = 3). (B, C) Representative images and statistics of intratumoral immunocytes, myeloid cells, B cells, T cells, CD8+ T cells, and CD4+ T cells between the WT and uPA–/– groups (n = 3). [file Image1.tif]

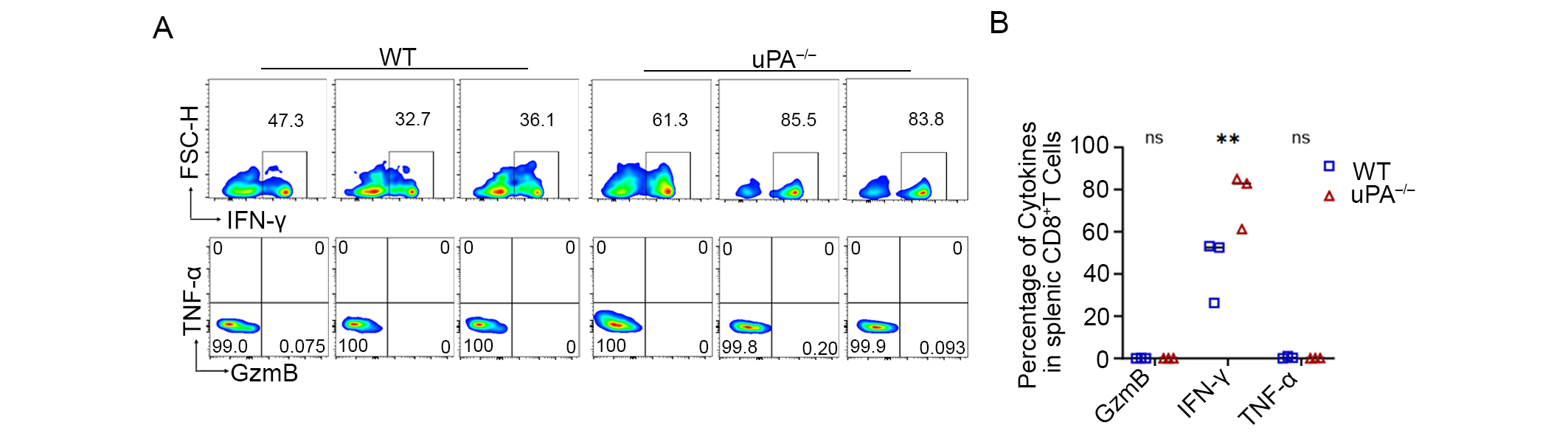

Supplement: Supplementary Figure 2 — uPA deficiency increases the expression of IFN-γ in splenic CD8+ T cells. (A) Representative flow cytometry plots showing the expression levels of cytotoxic molecules of T cells, such as TNF-α, IFN-γ, and GzmB, in Control and uPA–/– CD8+ T cells of the spleen (n = 3). (B) Percentage of GzmB, IFN-γ, and TNF-α in Control or uPA–/– CD8+ T cells (n = 3). [file Image2.tif]

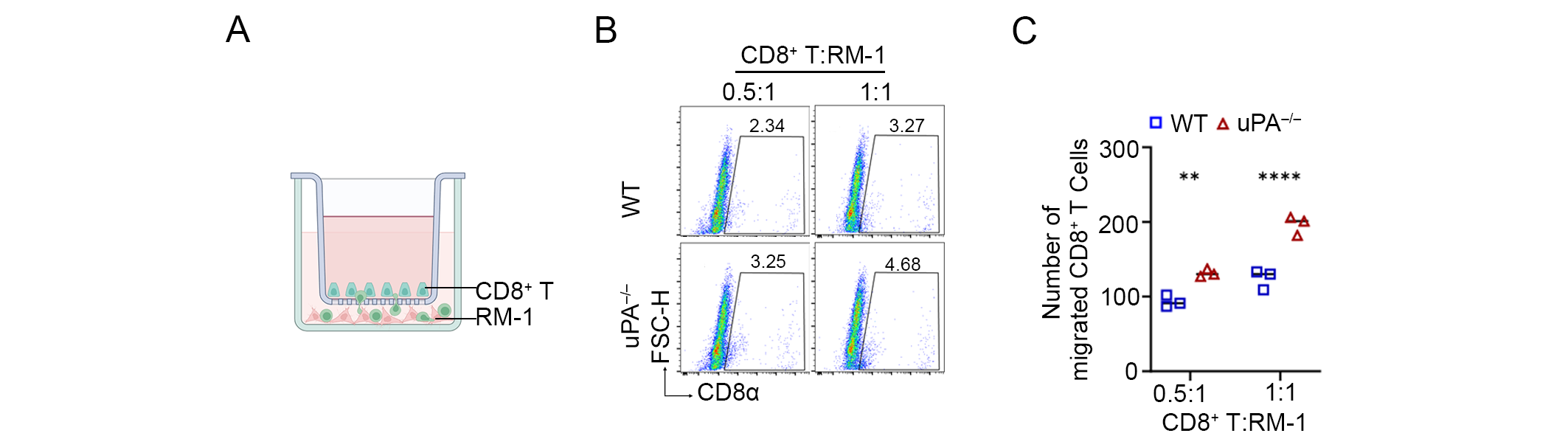

Supplement: Supplementary Figure 3 — Establishment and validation of WT CD8+CAR-T cells, uPA–/– CD8+CAR-T cells, and RM1-hCD19. (A) Representative flow cytometry plots showing the expression levels of CD19 CAR in WT and uPA–/– CD8+ CAR-T T cells. (B) Representative flow cytometry plots showing the expression levels of CD19 in RM-1 cells. [file Image3.tif]

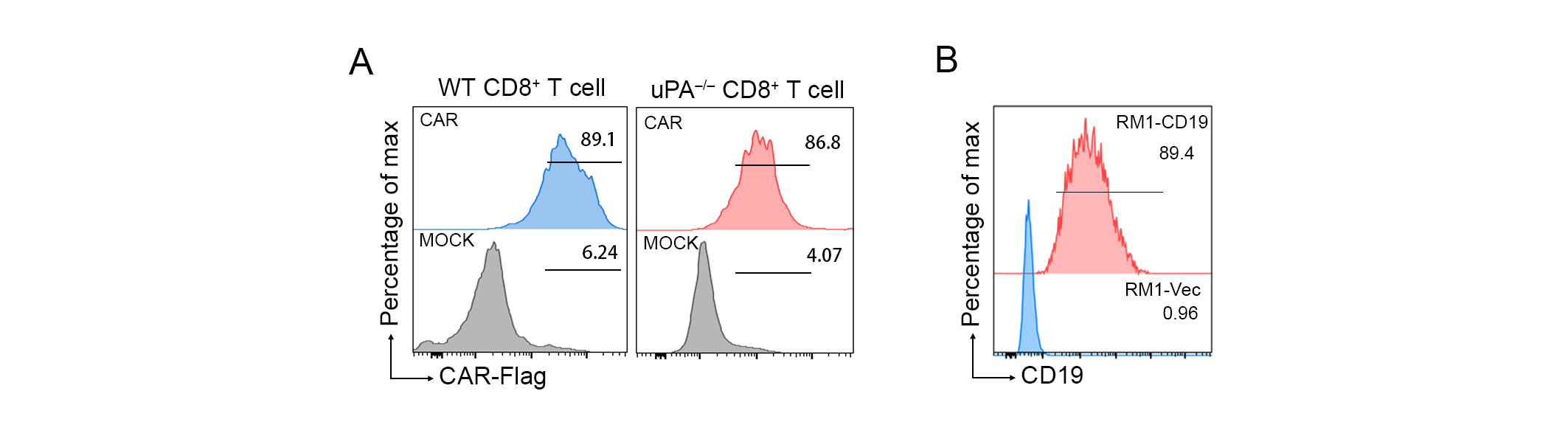

Supplement: Supplementary Figure 4 — uPA deficiency increases migrations of CD8+ T cells at different ratios. (A–C) Schematic diagram, flow cytometry plots, and statistics of migratory CD8+ T cells recruited by RM-1 cells after co-culturing at different ratios for 24 h (n = 3). [file Image4.tif]
